# Supplementary material for: Influenza Antibody Levels Associated with Laboratory-Confirmed Influenza in a Test-Negative Study Design, US Flu VE Network, November 2018–May 2019
Source: medRxiv. 2026 Mar 26:2026.03.24.26349239. Preprint. [Version 1] doi: 10.64898/2026.03.24.26349239 (PMC13042143; doi:10.64898/2026.03.24.26349239)
Supplement: Supplement 1 [file NIHPP2026.03.24.26349239v1-supplement-1.pdf]

Supplemental Table 1. Hemagglutination inhibition, microneutralization and neuraminidase inhibition assay antibody titers against antigens representing vaccine and circulating influenza viruses among test-negative control patients with acute respiratory illness by vaccination status.

Assay Antigen	**Vaccinated test-negative control patients N = 76**		**Unvaccinated test-negative control patients N = 54**	
	Geometric Mean Titer (95% CI)	Titer ≥40, n (%)	Geometric Mean Titer (95% CI)	Titer ≥40, n (%)
HI titer				
A/Michigan/45/2015-egg	102.4 (79.7, 131.5)	64 (84.2%)	29.6 (20.6, 42.5)	28 (51.9%)
A/Michigan/45/2015-cell	74.3 (57.1, 96.7)	61 (80.3%)	25.8 (18.3, 36.5)	25 (46.3%)
MN titer				
A/Singapore/INFIMH-16-0019/2016-egg	286.8 (215.8, 381.2)	71 (93.4%)	45.8 (30.2, 69.3)	30 (55.6%)
A/Kansas/14/2017-cell	33.3 (25.2, 43.8)	43 (56.6%)	11.6 (8.5, 15.9)	12 (22.2%)
NAI titer				
rH6N1	168.2 (129.0, 219.4)	70 (92.1%)	82.1 (58.7, 114.8)	40 (74.1%)
rH6N2	122.3 (98.6, 151.7)	71 (93.4%)	78.0 (55.5, 109.5)	41 (75.9%)

medRxiv preprint doi: https://doi.org/10.64898/2026.03.24.26349239; this version posted March 26, 2026. The copyright holder for this preprint (which was not certified by peer review) is the author/funder, who has granted medRxiv a license to display the preprint in perpetuity.
This article is a US Government work. It is not subject to copyright under 17 USC 105 and is also made available for use under a CC0 license.
